# Grapevine virus T diversity as revealed by full-length genome sequences assembled from high-throughput sequence data

**DOI:** 10.1371/journal.pone.0206010

**Published:** 2018-10-30

**Authors:** Shaheen Nourinejhad Zarghani, Jean Michel Hily, Miroslav Glasa, Armelle Marais, Thierry Wetzel, Chantal Faure, Emmanuelle Vigne, Amandine Velt, Olivier Lemaire, Jean Michel Boursiquot, Arnela Okic, Ana Belén Ruiz-Garcia, Antonio Olmos, Thierry Lacombe, Thierry Candresse

**Affiliations:** 1 DLR Rheinpfalz, Institute of Plant Protection, Neustadt an der Weinstrasse, Germany; 2 Department of Plant Protection, College of Abouraihan, University of Tehran, Tehran, Iran; 3 Université de Strasbourg, INRA, SVQV UMR-A 1131, Colmar, France; 4 Institute of Virology, Biomedical Research Centre, Slovak Academy of Sciences, Bratislava, Slovak Republic; 5 Equipe de Virologie, UMR 1332 BFP, INRA, Univ. Bordeaux, Villenave d’Ornon, France; 6 UMR 1334 AGAP, INRA, Montpellier SupAgro, Montpellier, France; 7 Centre de Ressources Biologiques de la Vigne, INRA, Marseillan-Plage, France; 8 University of Sarajevo, Faculty of Agriculture and Food Science, Sarajevo, Bosnia and Herzegovina; 9 Instituto Valenciano de Investigaciones Agrarias, Moncada, Valencia, Spain; Oklahoma State University, UNITED STATES

## Abstract

RNASeq or double-stranded RNA based approaches allowed the reconstruction of a total of 9 full-length or near full-length genomes of the recently discovered grapevine virus T (GVT). In addition, datamining of publicly available grapevine RNASeq transcriptome data allowed the reconstruction of a further 14 GVT genomes from five grapevine sources. Together with four GVT sequences available in Genbank, these novel sequences were used to analyse GVT diversity. GVT shows a very limited amount of indels variation but a high level of nucleotide and aminoacid polymorphism. This level is comparable to that shown in the closely related grapevine rupestris stem pitting-associated virus (GRSPaV). Further analyses showed that GVT mostly evolves under conservative selection pressure and that recombination has contributed to its evolutionary history. Phylogenetic analyses allow to identify at least seven clearly separated groups of GVT isolates. Analysis of the only reported PCR GVT-specific detection primer pair indicates that it is likely to fail to amplify some GVT isolates. Taken together these results point at the distinctiveness of GVT but also at the many points it shares with GRSPaV. They constitute the first pan-genomic analysis of the diversity of this novel virus.

## Introduction

Grapevine (*Vitis vinifera*) is one of the most important fruit crops with a production of more than 70 million metric tons/year worldwide, according to the last data released by the Food and Agriculture Organization of the United Nations (FAO) (www.fao.org/faostat; accessed July 2018). The grapevine industry is threatened by a wide range of pathogens, including a range of viruses associated to the four major viral diseases, infectious degeneration and decline, leafroll, rugose wood and fleck [1]. Recently, the application of high throughput sequencing (HTS) to plant virology has allowed the discovery of new viruses without prior information of their genome [2], increasing our knowledge on the diversity of the grapevine virome. There are currently over 70 plant viruses reported to infect grapevine, including members of the genus *Foveavirus* [1, 3]. Foveaviruses belong to the family *Betaflexiviridae* [4] and have helically filamentous particles ca. 800 nm long. These viruses have a positive sense, single-stranded, polyadenylated RNA genome, ranging from 8.4 to 9.3 kb in size. They have a single type of coat protein with a size of 28 to 44 kDa [3, 4]. Their genome harbors five open reading frames (ORFs), encoding from 5’ to 3’ a replication-associated protein (ORF 1), the putative movement proteins (ORF 2 to 4, constituting a triple gene block), and the coat protein (ORF 5), respectively [3, 4, 5]. Foveaviruses have no identified vectors and their transmission occurs by the vegetative propagation of their host plants [3]. Grapevine rupestris stem pitting-associated virus (GRSPaV), the type member of the *Foveavirus* genus, is one of the most prevalent viruses of grapevine [6–9]. Although the pathological properties of GRSPaV infection in most grapevine varieties remain largely unknown, it is consistently associated with ‘‘Rupestris stem pitting”, a component of the important Rugose wood complex of diseases [9–13]. GRSPaV exhibits extensive genetic diversity and may have evolved from an ancient recombination event between a Carlavirus and a Potexvirus [14]. Phylogenetic analyses of viral sequences derived from infected grapevines suggest that GRSPaV comprises a wide range of sequence variants [6–9, 15–18], which lead to the definition of four groups of sequence variants that can be further sub-divided in a number of subgroups [9, 18–19].

Recent advances in HTS technologies and bioinformatics have generated huge new opportunities for discovering and diagnosing novel plant viruses and viroids [2, 20]. Among the agents recently described in grapevine, Grapevine virus T (GVT) is a putative new member of the genus *Foveavirus*, closely related to GRSPaV. It was recently described from transcriptomic data of the Teroldego variety [21]. Since then, GVT has been reported from grapevines in Germany [22], Croatia [23] and Slovakia and the Czech Republic, where it appears to be relatively widespread [24]. Furthermore, similarly to GRSPaV, GVT seems to exhibit extensive genetic diversity [24]. A phylogenetic analysis performed on the complete GVT CP gene of several Slovakian and Czech isolates, together with the initial Teroldego isolate, has shown the existence of two well supported clusters of isolates and suggested the existence of potential additional phylogenetic clusters [24]. Taken together the data available on this recently described grapevine virus indicate significant prevalence and diversity, raising issues on its detectability and highlighting the need to generate more information on GVT diversity.

In the present work a total of 23 full-length or near full-length GVT sequences have been obtained by HTS analyses or more classical means. All these sequences, together with the few sequences already available in databases, have allowed us to perform an analysis of GVT diversity at the pangenomic level. The results presented here provide a first comprehensive picture on the genomic features and evolution of GVT, which might have important implications for the diagnostics of this recently discovered *Foveavirus*.

## Materials and methods

### Virus isolates and plant samples

The grapevine samples used in the present study are described in Table 1, which presents data on the variety, the sampled plants origin and the other viruses found to be present in these plants in addition to grapevine virus T.

**Table 1:** GVT isolate, grapevine variety, origin and tissue sampled for the grapevine plants analyzed here and viruses identified in addition to GVT.

GVT isolate	Variety^1^	Origin	Country^2^	Tissue sampled	Other viruses present	HTS template
**Limniona V34**	Limniona (2288Mtp2)	Germplasm collection^3^	France (Greece)	Phloem scrapings	GRSPaV, GLRaV2, GLRaV3, GFkV, GVA, GRVFV^4^	dsRNA
**Saperavi V49-12**	Saperavi (1734Mtp2)	Germplasm collection^3^	France (Romania)	Phloem scrapings	GRSPaV, GLRaV3, GVA, GVF, GRVFV^4^	dsRNA
**Saperavi V49-24**						
**Sauvignon gris V51**	Sauvignon gris (301Mtp4)	Germplasm Francetion^3^	France (Chile)	Phloem scrapings	GRSPaV, GPgV, GRGV^4^	dsRNA
**Sauvignon I27**	Sauvignon	MulFranceation block	France	Phloem scrapings	GRSPaV, HSVd, GYSVd1	Total RNA
**Sauvignon I60-2**				Phloem scrapings	GRSPaV, HSVd, GYSVd-1	Total RNA
**Sauvignon F79**				Leaves	GRSPaV, HSVd, GYSVd-1	Total RNA
**SK809**	Welshriesling	Commercial vineyard	Slovak Republic	Phloem scrapings	GLRaV-1, GRVFV, HSVd	rRNA depleted total RNA
**486–1**	Riesling	Commercial vineyard	Germany	Leaves	GRGV	na

^1^: the code of the accession is given in parentheses for samples originating from The INRA Vassal-Montpellier grapevine biological resource center.

^2^: the country of origin of the accession is given in parentheses for samples originating from a biological resource center.

^3^: INRA Grapevine biological resource center of Vassal-Montpellier (https://www6.montpellier.inra.fr/vassal_eng/)

^4^: potential presence of viroids not efficiently detected through the double-stranded RNA sequencing strategy used.

### High-throughput sequencing of cDNAs derived from highly purified double stranded RNAs

Highly purified double stranded RNAs were extracted from grapevine phloem scrapings of dormant canes using the small scale procedure described in [25]. This procedure involves two rounds of cellulose affinity chromatography and a nuclease digestion step to eliminate DNA and single-stranded RNAs. Complementary DNA molecules were then synthesized and randomly amplified, again as described [25, 26] before being sequenced on an Illumina HighSeq 2500 (2*250 nt paired-end reads) in a multiplexed format (Genewiz, South Plainfield, NJ, USA).

### High-throughput RNAseq

Total RNAs were extracted from phloem scrapings or from leaves, using the Spectrum Plant Total RNA Kit (Sigma). For the SK809 sample, ribosomal RNA was removed using the Ribo-Zero rRNA Removal Kit (Illumina, San Diego, USA) while total RNAs from other samples were directly converted to double-stranded cDNA from which sequencing banks were prepared. High-throughput sequencing in multiplexed format was performed on the following Illumina platforms: MiSeq (2*200 nucleotides) for the SK809 sample, HighSeq3000 (2*150 nt) for the three Sauvignon samples.

### High-throughput sequence data processing

Following demultiplexing and quality trimming, high-quality reads were used for *de novo* assembly and contigs were annotated by BlastN and BlastX analysis [27] against Genbank or by alignment to the viral genomes database (ftp://ftp.ncbi.nih.gov/genomes/Viruses/all.fna.tar.gz) using CLC Genomics Workbench 7.5 (http://www.clcbio.com) and Geneious v.8.1.9 (https://www.geneious.com/). In some cases, contigs were further extended by several rounds of mapping of residual reads in CLC Genomics Workbench. Subsequently, in order to assess the representation of GVT reads, the reads from each sample were mapped against the complete GVT sequence(s) assembled from that sample.

### Completion and validation of GVT genomic sequences assembled from the high-throughput sequence data

For some genome regions, the accuracy of the sequence of the assembled GVT genomic sequences was verified by direct Sanger sequencing of PCR products using primers designed from the NGS-based sequences. The same strategy was used to completely resequence and validate the sequence of the 486–1 isolate. Similarly, approximately 0.7 to 1.3 kilobases which were missing from the 5’ end of contigs assembled from double-stranded RNA data (isolates Limniona V34, Saperavi V49-12 and V49-24 and Sauvignon gris V51, see Table 2) were determined by direct Sanger sequencing of PCR products obtained using primers designed from the contigs and from available GVT sequences.

**Table 2:** GVT contig length, average coverage and representation in the dsRNA derived high-throughput sequencing reads derived from the various grapevine samples.

	Total reads	GVT Mapped reads	% mapped reads	Average coverage	Initial contig length	Final sequence length	Accession number
**Limniona V34**	716,447	9,890	1.38%	188 x	7.9 kb	8,670 nt	MH674173
**Saperavi V49-12**	427,125	9,389	2.2%	205 x	7,5 kb	8,670 nt	MH674171
**Saperavi V49-24**		5,318	1.25%	112 x	7,6 kb	8,670 nt	MH674172
**Sauvignon gris V51**	3,025,587	13,630	0.45%	322 x	7,3 kb	8,669 nt	MH674174

Terminal 3’ sequences were determined for all isolates (with the exception of Sauvignon I60-2 and F79) by direct Sanger sequencing of PCR products obtained using a polyA-anchored PCR and an internal primer designed from the contigs. For isolates Sauvignon I27 and 486–1, terminal 5’ sequences were determined using the 5’ Rapid Amplification of cDNA Ends (RACE) strategy and internal primers designed from the contigs, following the kit supplier’s instructions (Takara Bio Europe/Clontech, Saint-Germain-en-Laye, France).

### Determination of the complete genomic sequence of GVT isolate 486–1 by classical Sanger sequencing

For the 486–1 isolate, total RNAs were extracted from leaf material using the Nucleospin RNA plant and fungi kit (Macherey Nagel). The GVT genome was completely sequenced by Sanger sequencing of cDNA clones of overlapping PCR products, including for the 3’ terminal sequence, for which a polyA-anchored PCR was used. The 5’ terminal sequence was determined 5’ RACE (Invitrogen), following the instructions of the supplier. The cDNAs were dC- and dG-tailing in separate reactions, and amplified by PCR using a GVT-specific internal primer and oligo-dG or oligo-dC primers, respectively. At least four clones from each reaction were sequenced. The obtained 5’end sequences were all identical.

### Datamining of grapevine RNASeq data and assembly of GVT genomic sequences

All analyses were performed using the CLC Genomics Workbench 11.0 software. A selection of grapevine RNAseq transcriptome data available in GenBank (choice based on variety, country…) was downloaded and screened for the presence of GVT by a direct mapping of the reads to the GVT-Teroldego reference (NC_035203). Transcriptomes for which GVT presence was detected in this way were then assembled *de novo* using the following parameters: word size 19, bubble size 50 and a minimal contig size of 300nt. GVT contigs identified by BlastN analysis were then further extended by multiple rounds of residual reads mapping, until near complete genomes were obtained.

### Selection of a dataset of representative GRSPaV genomic sequences

A dataset composed of 21 full length or near full-length GRSPaV sequences selected so as to represent the known genetic diversity of GRSPaV was retrieved from the Genbank database. In particular these GRSPaV sequences were selected to be representative of the various phylogenetic groups and sub-groups identified in its diversity [6, 9, 18]. In total, group 1 is represented by four isolates including GRSPaV-SY, group 2 by 13 isolates (including GRSPaV-1 typifying subgroup 2a, GRSPaV-SG1, typifying subgroup 2b and GRSPaV-BS, group 3 by three isolates each typifying a subgroup (GRSPaV-JF, GRSPaV-ML and GRSPaV-PN) and group 4 by the divergent isolate GRSPaV-LSL.

### Sequence, diversity and phylogenetic analyses

Multiple sequence alignments were performed using ClustalW [28] implemented in MEGA version 7 [29]. Phylogenetic trees were constructed using the neighbor-joining method with strict nucleotide or amino acid distances and randomized bootstrapping for the evaluation of branch validity. Genetic distances (p-distances calculated on nucleotide or amino acid identity) were calculated using MEGA version 7. An analysis of the local diversity along the GVT genome was performed using DNASP v6 [30] while a search for potential recombination events in GVT genomic sequences was performed using RDP 4.0 [31]. A codon-based analysis of selection pressures in the various GVT open reading frames was performed using the FEL and SLAC programs on the Datamonkey web server (http://www.datamonkey.org/)[32].

## Results and discussion

### Determination of the complete genome sequence of GVT isolates using RNAseq or dsRNA high-throughput sequencing approaches

As indicated in the Materials and Methods section, in the course of the analysis of the grapevine virome a number of samples were analysed by high-throughput sequencing (HTS), either by a double-stranded RNA (dsRNA)-based approach or by a total RNA or ribosomal RNA-depleted RNASeq approach. In parallel, the GVT genome of isolate 486–1 was completely determined by classical Sanger sequencing. In total, GVT was identified in eight samples, three of which represent the same Sauvignon origin. Besides these three Sauvignon samples from France, were three accessions (Limniona (2288Mtp2), Saperavi (1734Mtp2), Sauvignon gris (301Mtp4) obtained from the INRA Grapevine biological resource center of Vassal-Montpellier (https://www6.montpellier.inra.fr/vassal_eng/), a Welshriesling sample from Slovakia and a Riesling sample from Germany (Table 1). All of these samples were found to be simultaneously infected by several other viruses and, in most cases, viroids (Table 1). However, some of these samples (French Sauvignon, Slovak Welshriesling and German Riesling) did not show any symptoms despite the mixed infections affecting them.

Following the bioinformatics analysis of the sequencing reads of the three grapevine samples analysed by the dsRNA-based approach (Table 2), large GVT contigs were assembled for the Limniona and Sauvignon gris samples while two GVT contigs were assembled from the Saperavi sample (Table 2). As compared to the reference GVT genome (NC_035203)[21], these large contigs (7.3 to 7.9 kilobases, see Table 2) were missing approximately between 0.7 to 1.3 kb at their 5’ end and 72–108 nucleotides at their 3’ end. They all however had an otherwise excellent average coverage of between 112x and 322x (Table 2). The 3’ sequences of the various isolates were completed by the direct sequencing of PCR products amplified using a specific primer designed from the initial contig and an oligodT anchored primer. The 5’ sequences were completed by the direct sequencing of PCR products using specific primers designed from the initial contig and a primer designed from the 5’ end of I27 GVT sequence. In all cases, the overlap regions between the initial contig and the PCR products were found to be 100% identical. No effort was made to resequence the region covered by the 5’ terminal amplification primer, which was therefore not integrated in the finalized sequences.

In parallel, GVT genomes were similarly assembled from high-throughput Illumina data derived from total RNAs or from ribosomal RNA-depleted total RNAs (Table 3). In the case of the three Sauvignon samples, near complete contigs were readily assembled, missing only 14–18 nt at the 5’ end and 23–27 nt at the 3’ end as compared to the NC_035203 reference isolate. These three contigs originate from the same grapevine variety and are highly homologous, sharing a minimum of 99.1% nucleotide (nt) identity (the sequences assembled from the I60-2 phloem scrappings and the F79 leaves are in fact 100% identical). It was therefore decided to complete the sequence of a single variant, I27. This was achieved as described above for the 3’ terminal region and by a RACE PCR for the 5’ terminal region. The completed genome of the I27 isolate is 8,694 nt long, excluding the 3’ polyA tail. The genomes of the SK809 isolate was similarly assembled, while that of the 486–1 isolate was fully determined by the Sanger sequencing of overlapping PCR products. In the case of the SK809 isolate, the genome was partially resequenced in some regions by direct Sanger sequencing of PCR products. No effort was made to complete the nine missing terminal 5’ nucleotides but the 3’ end was completed as described above. For the 486–1 isolate, the 5’ and 3’ terminal regions were determined by RACE and polyA-anchored PCR. Similar to the I27 isolate, the genomic sequence of the 486–1 isolate is 8,694 nt in length, excluding the polyA tail, a value to be compared to the 8,701 nt of the reference NC_035203 isolate.

**Table 3:** GVT contig length, average coverage and representation in the total RNA derived high-throughput sequencing reads derived from the various grapevine samples.

Isolate	Total reads	Reads after grapevine genome substraction	GVT mapped reads	% mapped reads	Average coverage	Accession number
**I27**	70,808,936	15,942,747	575	0.003%	9.9 x	MH674170
**I60-2**	57,440,410	10,894,109	2,459	0.023%	42.3 x	MH674175
**F79**	73,210,304	9,338,200	4,336	0.046%	74.8 x	MH674176
**SK809**	1,065,468	na^1^	2,532	0.23%	36.6 x	MH638265

^1^: does not apply.

### Datamining of grapevine RNAseq transcriptomic data allows the assembly of near complete GVT genomes

In an attempt to identifiy and assemble additionnal complete or near complete GVT variant genomic sequences, publicly available grapevine transcriptomic RNAseq data were screened for the presence of GVT by mapping reads against the GVT reference sequence using CLC Genomics Workbench 11.0. In order to allow the detection of the broadest GVT diversity possible, relaxed parameters for length fraction (0.5) and similarity (0.7) were used while default values were used for other parameters. GVT genomes were then recovered from GVT-positive datasets by *de novo* assembly. Contigs were finally extended by multiple rounds of mapping and assembly as described recenty in the case of GRSPaV [18]. In total, 14 new near complete GVT genomes could be assembled in this way (Table 4) from six different grapevine varieties.

**Table 4:** Dataset, grapevine variety and length of the GVT contigs assembled with datamining publicly available grapevine transcriptomic data.

Isolate	Dataset	Grapevine species/ variety	Length of contig	% of GVT genome	Accession number
Pinot noir	SRR520387	*V*. *vinifera* cv. Pinot Noir	8,679 nt	99.7%	MH674167
Barbera	SRR1631833	*V*. *vinifera* cv. Barbera	8,665 nt	99.6%	MH674169
Moscato rosa	ERR923384	*V*. *vinifera* cv. Moscato rosa	8,667 nt	99.6%	MH674169
CF-1	ERR922632	*V*. *vinifera* cv. Cabernet franc	8,672 nt	99.7%	MH674178
CF-2		*V*. *vinifera* cv. Cabernet franc	8,674 nt	99.7%	MH674179
CF-3		*V*. *vinifera* cv. Cabernet franc	8,657 nt	99.5%	MH674180
CF-4		*V*. *vinifera* cv. Cabernet franc	8,637 nt	99.3%	MH674181
Lambrusco-1	ERR922631	*V*. *vinifera* cv. Lambrusco	8,687 nt	99.8%	MH674182
Lambrusco-2		*V*. *vinifera* cv. Lambrusco	8,635 nt	99.2%	MH674183
Lambrusco-3		*V*. *vinifera* cv. Lambrusco	8,319 nt	95.6%	MH674184
Lambrusco-4		*V*. *vinifera* cv. Lambrusco	8,683 nt	99.8%	MH674185
Lambrusco-5		*V*. *vinifera* cv. Lambrusco	8,651 nt	99.4%	MH674186
Lambrusco-6		*V*. *vinifera* cv. Lambrusco	8,557 nt	98.3%	MH674187
Vitis amurensis	SRR922004, SRR922126, SRR922136, SRR922138	*V*. *amurensis*	8,668 nt	99.6%	MH674168

Pinot noir, Barbera, Moscato rosa and the wild grapevine relative *Vitis amurensis* each yielded a single variant while respectively four and six variants could be assembled from Cabernet Franc and Lambrusco, respectively. Only in the case of *V*. *amurensis*, for which GVT coverage was lower, was it necessary to perform an assembly from multiple datasets. However, there was no evidence for GVT variability between the assembled datasets. Since these genomes were obtained by datamining, no specific efforts were made to complete the missing 5’ or 3’ terminal sequences. The amount of missing data is however limited since only 4–29 nt are missing from the 5’ end of the assembled genomes as compared to the NC_035203 reference isolate, while 4–53 nt are missing from the genomes 3’ ends. Only for one variant, Lambrusco-3 did a more significant 3’ region end up missing (363 nt). Overall, as compared to the reference isolate, the assembled contigs represent over 99.2% of the complete genome (99.2–99.8%) with the exception of the Lambrusco-6 (98.3%) and of the Lambrusco-3 isolates (95.6%) (Table 4).

### Identification of GVT terminal genomic sequences

In total, 8 new complete or near-complete GVT genomes assembled from high-throughput dsRNA or RNAseq data (Tables 2 and 3) and a complete genome determined by Sanger sequencing were thus obtained, together with another 14 genomes assembled from publicly available grapevine RNAseq transcriptome data (Table 4). These were compared to the reference isolate, to the two incomplete sequences reported from Croatian autochtonous grapevines [23, MG001925-26] and to a complete, unreleased sequence of a Slovak isolate from the Veltliner variety [24, MH182704]. The reference sequence has been assembled from transcriptomic data of the Teroldego variety but not experimentally validated [21]. The precise 5’ end of the GVT genome has thus been determined only in three isolates, the MH182704 sequence [24] and the I27 and 486–1 isolates reported here. The sequence determined for these last two isolates are identical (5’ GGATAAAC…) while the MH182704 and NC_035203 sequences are further extended 5’ but by different sequences: **acatggg**GGATAAAC…. (the extension in bold lower case) for MH182704 and **cgc**GGATAAAC…. for the NC_035203 reference sequence. Given these discrepancies it is not possible to unambiguously conclude on GVT 5’ genome end but the convergence of the results independently obtained on the I27 and 486–1 suggests that the extreme 5’ end has been identified. The possibility that different isolates might have different 5’ terminal sequences remains but seems unlikely in view of the high conservation of these sequences between isolates in most viruses. The absolute conservation of the terminal 19 5’ nucleotides in all isolates of the closely related GRSPaV [9] also argues aginst this notion. Assuming the terminal sequence identified in the I27 and 486–1 isolates to be correct, the first 16 nucleotides would be fully conserved between all GVT isolates sequenced here: 5’ GGATAAACAACAGAAC 3’. This sequence shows high homology with the corresponding GRSPaV sequence, which only differs by a single terminal G and by the insertion of the ATAAC sequence in bold and underlined here: 5’ GATAAAC**ATAA**CAACAGAA 3’.

In all the isolates analysed here, highly homologous 3’ terminal sequences were identified. Surprisingly the sequence of the NC_035203 GVT reference isolate is four nt longer and completely unrelated to the sequence of all other isolates for its last 21 nucleotides. Given the expected high conservation of the 3’ terminal sequence (this region is again highly conserved between GRSPaV isolates [9]) and the fact that the reference sequence has not been experimentally confirmed, the most likely explanation is that the reference sequence contains an assembly artefact. The alternative possibility, that the reference isolate possesses a highly divergent 3’ terminal sequence, although less likely, cannot however be totally disproved. But overall a change in the choice of the GVT reference isolate for an isolate with experimentally validated genome ends seems warranted.

### GVT genomic organization is highly conserved between isolates

The genomic organization is fully conserved between all GVT isolates and is typical of members of the *Foveavirus* genus [3, 4], with its five open reading frames (ORFs). From 5’ to 3’, these correspond to a large replication-associated protein (REP, ORF1), three ORFs encoding the three proteins of a movement-associated triple gene block (TGB1-2-3, ORFs 2-3-4)[5] and a coat protein (CP, ORF5). The genome is highly colinear between all isolates, with very few indels. The MG001925 sequence contains a six nt [two amino acids (aa)] deletion in REP gene and a two nt (frameshift) deletion in CP gene. However, the genome coverage for this isolate is incomplete so that it may still contain some errors. No other indels in the genome coding regions are identified among the 27 genomic sequences analyzed here.

Minor indel polymorphisms are observed in the internal non-coding region (NCR) between the end of REP ORF and the beginning of the TGB1 ORF. The Lambrusco-1 has three extra nt in that region while the Lambrusco-3 and CF-4 isolates each have a one nt deletion. A single one nt deletion is observed in the 3’NCR in the Sauvignon gris V51, CF-4 and Lambrusco-4 and Lambrusco-5 isolates.

The REP ORF is conserved in length at 6,387 nt (2,129 aa) and ends with TAG stop codon (TGA for Lambrusco-1). The encoded protein contains all the expected conserved domains: Methyltransferase (pfam01660, aa 43–354), AlkB (COG3145, aa 721–849), Peptidase C23 (pfam05379, aa 1155–1239), Helicase 1 (pfam01443, aa 1332–1590) and RdRp 2 (pfam00978, aa 1721–2116). These motives are similarly positionned and spaced as in the GRSPaV REP, with the exception of a ca. 30 aa longer region between the Methyltransferase and AlkB domains.

The TGB1 ORF is fully conserved in length (663 nt, 221 aa) and ends with TGA or TAA stop codons. The TGB2 ORF is conserved in length (348 nt, 116 aa) in all isolates except Limniona V34 (357 nt, 119 aa, three aa longer) because of a mutation changing the conserved TGA stop codon in an AGA and termination on the next in-frame stop codon (TAG), for a three aa longer protein. The TGB3 is the most variable ORF size-wise. It is generally 234 nt long (78 aa) but is 246 nt (82 aa) in 486–1 and 249 nt (83 aa) in the three Sauvignon variants I27, I60-2 and F79. In each case of a longer protein, termination is on the next in-frame stop codon, four or five amino acids downstream. Depending on the isolate, TGB3 terminates, on TGA, TAG or TAA stop codons. The expected conserved motives are found in each of the TGB proteins: Viral_helicase1 (pfam01443, TGB1 aa 25–220), Plant_vir_prot (pfam01307, TGB2 aa 4–62) and the misnamed 7kD_coat (pfam02495, TGB3 aa 6–60).

The CP ORF is fully conserved in length, at 765 nt (255 aa) and ends either with TGA, TAA or TAG stop codons. It contains the expectyed Flexi_CP conserved motif (pfam00286, aa 72–209).

**Fig 1:**
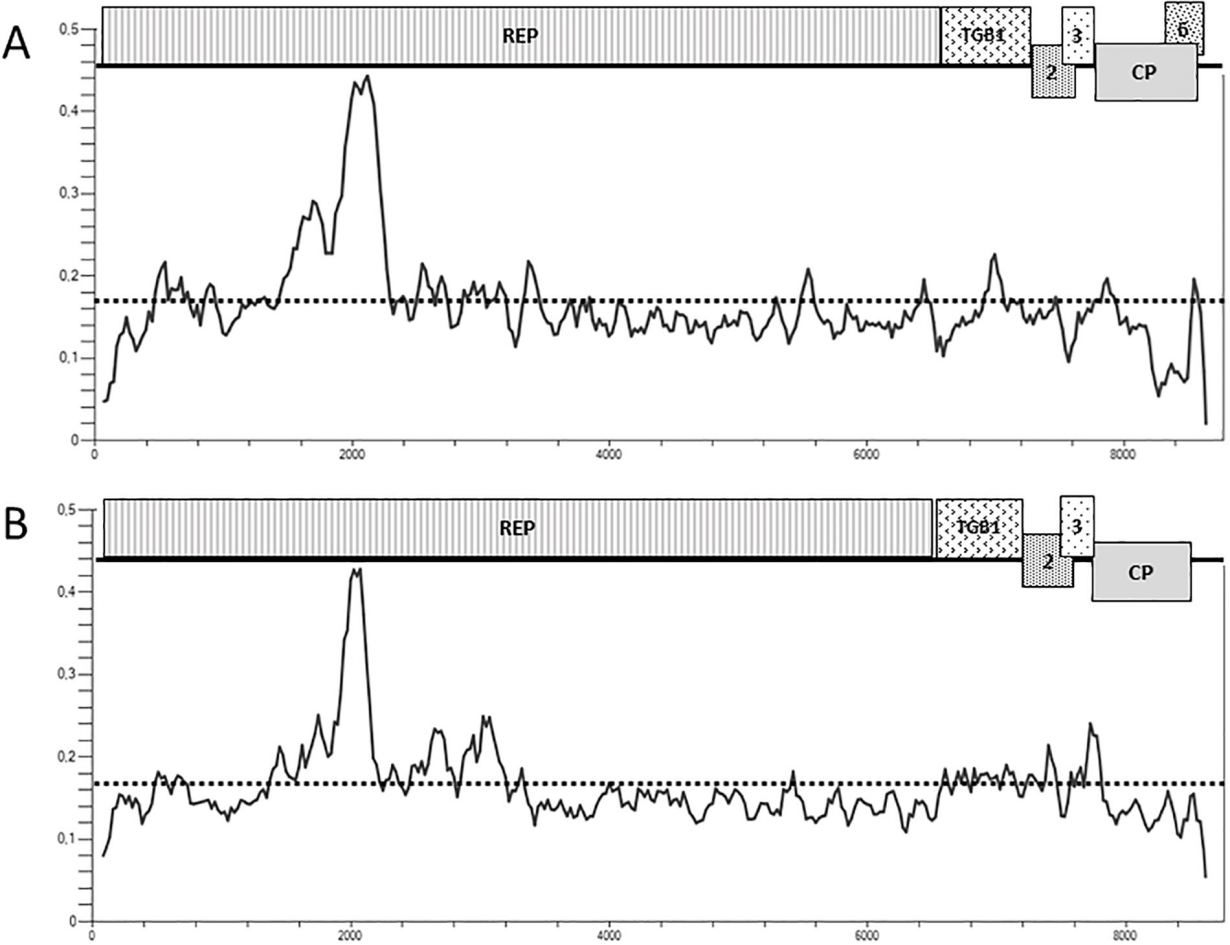
Schematic representation of the genomic organization of GRSPaV (A) and of GVT (B) and plotting of their respective nucleotide diversity (pi) along their genomes. Open reading frames are indicated by boxes. REP: replication-associated protein; TGB1, 2, 3: Triple gene block protein 1, 2, 3; CP: coat protein; 6: ORF6. The overall average diversity calculated for the complete genome is indicated by a doted line. Pi plots were constructed using DNASP v6, with a window of 100 nt and a step of 25 nt.

Overall this genomic organization is highly parallel to that of the closely related GRSPaV (Fig 1) although GRSPaV has an additional putative ORF that largely overlaps with the 3’ part of the CP gene [9, 13]. In some GRSPaV isolates this ORF encodes a protein of up to 14 kDa, but if assuming translation initiation on a conserved methionine, this conserved extra ORF encodes a 95 aa protein. This potential protein has no homology with any other protein in Genbank. This putative ORF is not observed in any GVT isolate. Although a similar placed, but shorter potential ORF can be observed in some apple stem pitting virus (ASPV) isolates, it is not conserved in ASPV and it is not observed in other foveaviruses such as rubus canadensis virus 1, peach chlorotic mottle virus, asian prunus virus 1 or apricot latent virus. This fully conserved ORF appears thus to be a feature so far unique to GRSPaV in the *Foveavirus* genus and to differenciate it from GVT.

### Analysis of GVT variability using full genome sequence data

Using the dataset of 27 full length or near full length GVT sequences we then analyzed GVT diversity. At whole genome level, the average pairwise divergence between GVT isolates was calculated to be 16.6% +/- 0.2%. This relatively high value is in line with the known high variability of many *Betaflexiviridae* members and is comparable to the 16.2% +/- 1.9% computed for a dataset composed of 21 full length or near full-length GRSPaV sequences selected so as to represent the known genetic diversity of GRSPaV [6, 9]. Remarkably, at respectively 17.3% +/- 0.3% and 17.7% +/- 0.2%, the average genetic distance between the various isolates identified in the Cabernet franc or Lambrusco transcriptomes proved as high as the overall GVT value, demonstrating the extreme GVT diversity involved in these mixed infections.

The GVT 5’ and 3’ NCRs are slightly more conserved as compared to the rest of the genome, with average divergence values of respectively 7.6 and 11.6% (Table 5). The CP ORF is the most conserved (14.8% +/- 0.7%), while the TGB1 is the most variable (17.3% +/- 0.8%), closely followed by the REP and TGB2 ORFs. Looking at the encoded proteins, somewhat different trends are observed. The CP is again the most conserved with only 6.1% average divergence. This high conservation of the CP is also observed in GRSPaV [9]. However the second most conserved protein is the REP (9.8% average divergence) followed in order by the TGB1 and TGB2, while at 17.9% the TGB3 appears to be the less conserved GVT protein. As previously shown for other *Betaflexiviridae* members [33] a very large fraction of GVT diversity is concentrated on the third position of codons which shows from three to six times more diversity than the first position of codons. The comparable values are even higher when considering the 2^nd^ position, which is the most constrained (Table 5).

**Table 5:** Average pairwise nucleotide (nt) and amino acid (aa) divergence computed for all genomic regions of GVT. The value computed individually for the different codon positions are also shown.

	Full genome	5’ NCR	REP	TGB1	TGB2	TGB3	CP	3’NCR
Average pairwise divergence (nt)	16.6 +/- 0.2%	7.6 +/- 2.3%	17.0 +/- 0.2%	17.3 +/- 0.8%	16.8 +/- 1.2%	15.7 +/- 1.5%	14.8 +/- 0.7%	11.6 +/- 1.1%
Avegage pairwise divergence (aa)	na	na	9.8 +/- 0.4%	11.9 +/- 1.2%	13.9 +/- 2.0%	17.9 +/- 2.6%	6.1 +/- 0.9%	na
Average pairwise divergence (1^st^ codon position)	na	na	8.7 +/- 0.3%	9.7 +/- 1.1%	13.3 +/- 1.8%	9.1 +/- 1.6%	5.7 +/- 0.9%	na
Average pairwise divergence (2^nd^ codon position)	na	na	4.8 +/- 0.2%	6.2 +/- 0.9%	4.3 +/- 1.1%	10.2 +/- 1.7%	3.5 +/- 0.5%	na
Average pairwise divergence (3^rd^ codon position)	na	na	37.5 +/- 0.3%	36.0 +/- 1.1%	32.8 +/- 1.9%	27.8 +/- 2.4%	35.1 +/- 1.1%	na

Plotting of the nucleotide diversity (pi) along the genome confirms these observations and shows a very comparable profile between GVT and the closely related GRSPaV. In particular, the two viruses show a steep diversity peak around position 2000 of the genome (Fig 1). This is immediately upstream of the AlkB domain found at positions 721–849 (aa) of the REP protein of GVT. The CP gene and the 3’ NCR tend for both viruses to show a lower diversity than the rest of the genome. However, this trend is clearly stronger in the case of GRSPaV with a very strong drop in diversity, which largely corresponds to the region of overlap between the CP gene and the tentative ORF6. In this region, which corresponds to nearly half of the CP gene, the GRSPaV nucleotide diversity is sharply lower (7.7 +/- 0.8%) as compared to the upstream portion of the gene (15.0 +/ 1.0%). This trend is particularly strong for the 2^nd^ position of the codons, for which diversity is reduced more than four-fold (from 2.7% to 0.6%) while codon positions 1 and 3 show only roughly a halving of the diversity, in line with the trend computed when considering all positions. This particular diversity pattern is coherent with the existence of a conservative selection pressure applying to ORF6 and therefore supports the hypothesis of the functionality of this GRSPaV ORF.

A codon-based analysis of selection pressures was performed on the five ORFs encoded on the GVT genome using the FEL and SLAC programs on the Datamonkey web server [32]. This analysis failed to identify any evidence for the existence of codons under divergent selection pressure at the 1% threshold, suggesting that GVT largely appears to be evolving in grapevine under conservative selection pressure.

### Phylogenetic analysis of GVT isolates demonstrates the existence of several clusters of isolates

**Fig 2:**
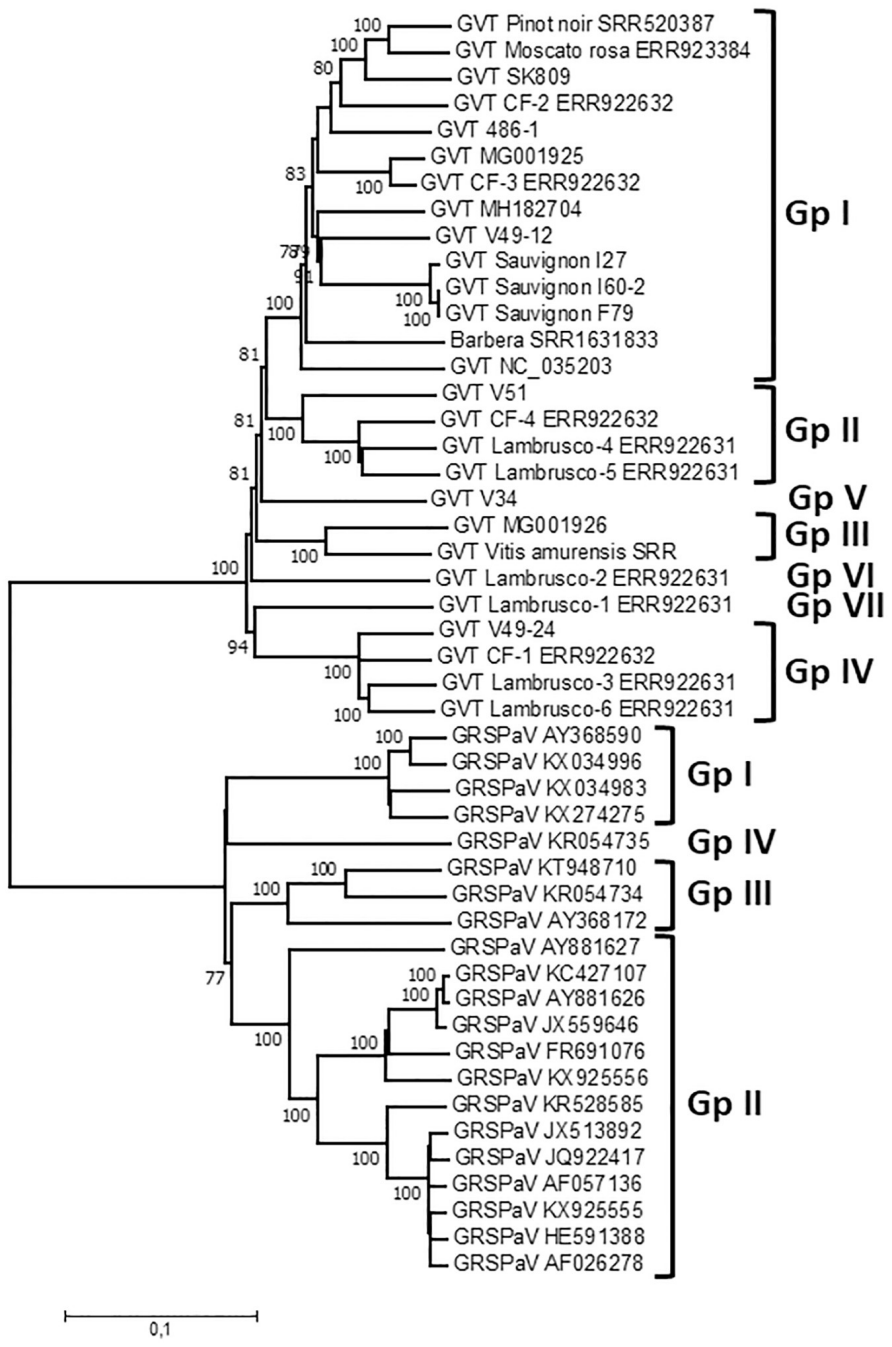
Phylogenetic tree reconstructed using the complete or near-complete genome sequence of GVT and GRSPaV isolates. The Neighbor joining tree was reconstructed using a strict nucleotide distance in Mega7. Bootstrap analysis (1,000 replicates) was performed to evaluate the solidity of branches. Only bootstrap values >70% are shown. The scale bar represents 10% nucleotide divergence. Groups of GRSPaV or GVT are indicated on the right of the figure.

A phylogenetic analysis of the complete or near complete GVT genome sequences was performed using the sequences reported here and those available in Genbank. The 21 GRSPaV genome sequences selected so as to represent its known genetic diversity were analyzed in parallel, providing a benchmark on which to compare GVT diversity. Fig 2 shows a very clear separation of GVT from GRSPaV, further supporting its recognition as a distinct *Foveavirus* species. For GRSPaV, the four well known major phylogenetic clusters are observed [9], with group IV represented by the divergent LSL isolate [34, KR054735]. The GVT portion of the tree similarly reveals four clusters of isolates supported by 100% bootstrap values (Fig 2). In addition, a further three groups correspond to single isolates at the end of long branches (Limniona V34, Lambrusco-1 and Lambrusco-2). These various clusters are quite distinct and clearly identified. They show an average intragroup divergence of between 7.4% +/- 0.2% (Group IV) and 12.2% +/- 0.2% (Group I) while the intergroup divergence varies between 17.3 and 19.8% +/- 0.4%. Comparable values for GRSPaV range from 5.5% +/- 0.2% (Group I) to 14.4% +/- 0.2% (Group III) for intragroup values but are slightly higher, between 22.7 and 23.4% +/-0.3% for intergroup values.

Additional analyses performed on the individual REP and CP ORFs at the nucleotide (not shown) or amino acid levels (S1 and S2 Figs) further confirm this clustering of GVT isolates in a number of well separated groups. For both GRSPaV and GVT, the intraspecific diversity is clearly narrower when considering the CP tree, thus confirming the diversity analysis performed on the individual ORFs. The detailed comparison of the REP and CP trees shows, however, a different behavior of isolate SK809 in the two trees: while it clusters with 100% bootstrap support in Group I in the REP tree, it clusters in Group II in the CP tree with significant bootstrap support (S1 and S2 Figs). As this type of behavior can be indicative of recombination events, a specific search for recombination events in the GVT full genomes dataset was performed.

### Search for potential recombination events in GVT isolates

The two Croatian GVT sequences [23] were removed from the dataset because they contain internal gaps and because their assembly might still contain artifacts. The remaining dataset of 25 near complete GVT sequences were analyzed using the RDP4 software [31]. Some statistically supported potential recombination events could be identified, involving two groups of isolates. In one case, four recombination events involving isolates 486.1, CF-3 and Moscato rosa are detected by seven of the nine tested methods, with combined corrected probabilities varying between 1.0 e^-7^ and 9.0 e^-26^ (Table 6). These events affect different parts of the genome and suggest the existence of multiple recombination events linking these isolates and a possible complex mosaic structure for the genome of one of them. The second set of tentative recombination events detected involve isolates SK809 and Moscato rosa and either an unknown parent or the Lambrusco-1 isolate. The highest probability event (involving Lambrusco-1, detected by 7/9 programs with an excellent overall corrected probability of 1.3 e-^37^) recapitulates the other two detected events, that have lower probabilities (Table 6).

**Table 6:** Potential recombination events detected by RDP4 in the genome of GVT isolates.

Predicted recombinant	Predicted major parent	Predicted minor parent	Detecting programs^1^	Predicted breakpoints	Overall corrected probability
486–1	CF-3	Moscato rosa	7	1538–2838	9.0 e^-26^
			7	3772–4104	1.0 e^-7^
			7	5888–6824	2.5 e^-10^
			7	7952–8624	3.3 e^-13^
SK809	Lambrusco-1	Moscato rosa	7	438–7770	1.3 e^-37^
	unknown	Moscato rosa	5	1952–2403	2.5 e^-5^
			6	3139–5299	1.3 e^-20^

^1^: number of programs that detected a particular potential recombination event out of the nine tested algorithms.

Taken together with the abnormal behavior of isolate SK809 between the REP and the CP phylogenetic tree, these results indicate that, similar to many other *Betaflexiviridae* members, recombination appears to have played a significant role in the evolution of GVT. This observation was not unexpected in view of the complex mixed infections of GVT isolates reported here and of the known impact of recombination on the evolution of the closely related GRSPaV [9, 17–18].

### Consequences of GVT variability for its molecular diagnostics

**Fig 3:**
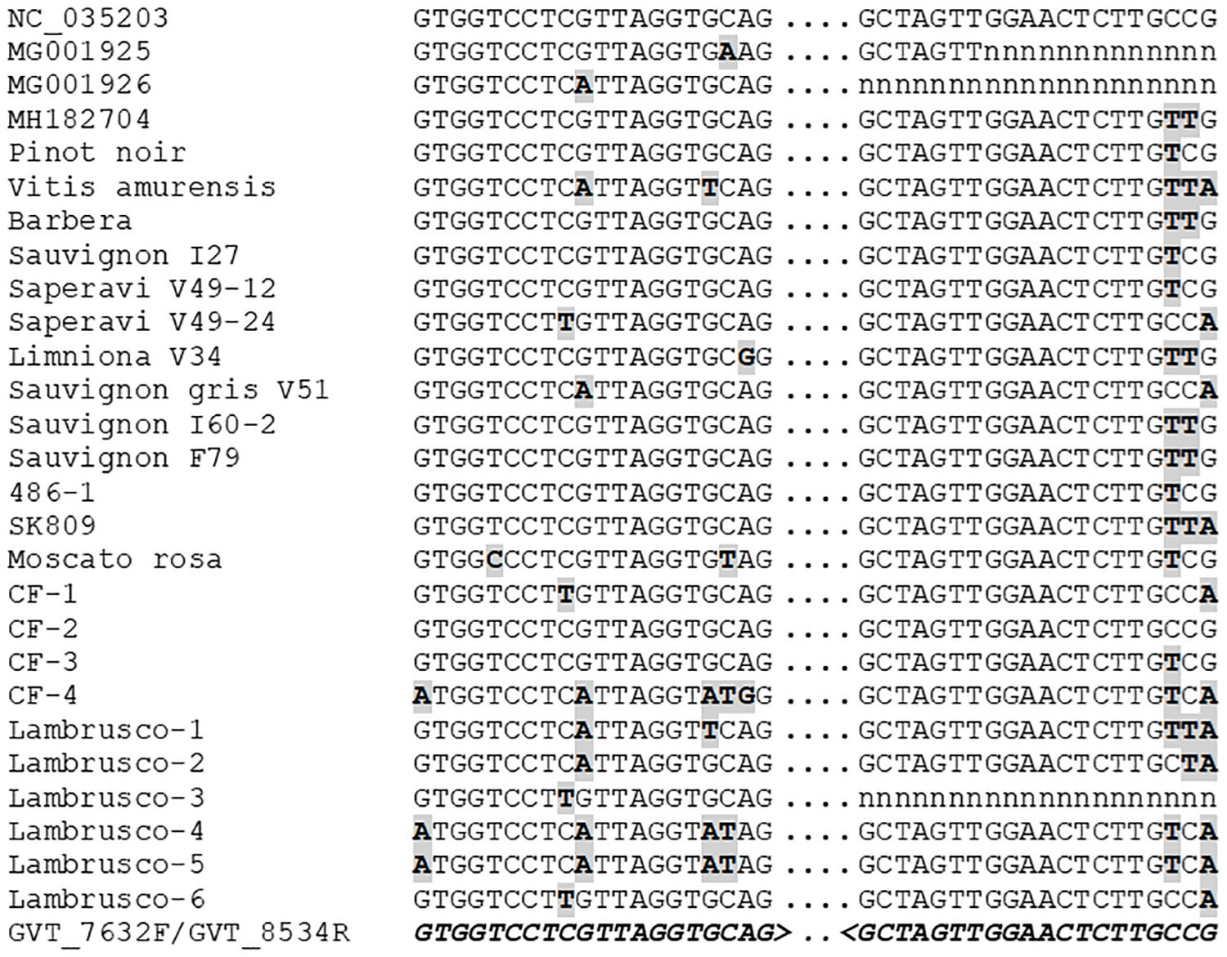
Comparison of the sequence of the various GVT isolates at the binding sites of the GVT_7632F/GVT_8534R detection primer pair [24]. The sequences are shown in a 5' to 3' orientation (left to right). Missing sequence information is indicated by n. Mutations are indicated in gray-shaded, bold type. The sequence of the primers is shown in bold italics at the bottom of the alignement (for the GVT_8534R reverse primer, the reverse complement is shown).

The diversity of GVT evidenced in the present work is quite large, as evidenced by the 16.6% average divergence between isolates at full genome level reported here. This translates in an average 3.3 differences for a 20 nucleotides PCR primer binding site, indicating that designing detection primers able to detect all GVT isolates might be a challenging task. To date, as single pair of PCR primers is reported for GVT [24]. In an attempt to evaluate the polyvalence of these primers, we confronted them to the 27 GVT genomes analyzed here. Of particular interest for this comparison is the fact that having been identified by high-throughput sequencing approaches, these isolates can be considered as an unbiased representation of GVT diversity. The results of this comparison are presented in Fig 3.

For the reverse primer GVT_8534R, the first 17 nucleotides from the 3’ primer end are fully conserved for all isolates for which sequence information is available in that region. The last three nucleotides of the primer match with from zero to three mutations with the sequence of the various isolates. However, these mutations are unlikely to drastically affect binding of the primer, which is therefore likely to show excellent polyvalence. In the case of the forward primer, GVT_7632F, the situation is somewhat different. From zero to five mutations affect the primer binding site, depending on the GVT isolate. In some cases, these mutations affect positions close to the primer 3’ end. This is particularly the case for the Lambrusco-1 isolate (five mutations, one of which is internal and three of which are clustered next to the primer 3’ terminal nucleotide) and, to a slightly lesser extent, of isolates Lambrusco-4 and -5 (four mutations, two clustered close to the primer 3’ end) (Fig 3). Although this would have to be experimentally confirmed, the number and critical position of the mutations in the primer binding site strongly suggest that the efficiency of the GVT_7632F primer is likely to be severely affected if not abolished for some GVT isolates.

## Conclusion

The sequences and analyses reported here represent the first in depth, full genome analysis of GVT diversity. The only information available to date on GVT diversity is a PCR-based analysis [24], which was limited to the CP gene of Czech and Slovak isolates. The authors report an average pairwise divergence of 11.2% +/- 0.8% at the nucleotide level and of 8.7% at the amino acid level. These values are close to those reported here (14.8% +/- 0.7% for the nucleotide sequences, 6.1% +/- 0.7% for the amino acid sequences), confirming these initial results.

GVT shares many features with its closest relative, GRSPaV, which is also a grapevine virus. In both cases, there is very little indel polymorphism, while single nucleotide polymorphism rates are high, in particular on the third positions of codons. The genetic organization of the two viruses are highly similar, with the exception of the presence of a conserved, putative ORF6 largely overlapping the CP ORF in GRSPaV, that is absent in GVT. GVT diversity appears to be relatively high and of the same order of magnitude than that of GRSPaV. Similar to GRSPaV, GVT diversity is structured and the phylogenetic analyses reported here allow the delineation of at least seven quite clearly separated groups of isolates. The recombination analysis performed here also provides the first evidence for recombination in GVT.

As for other viruses with a high diversity, the development of GVT PCR-based detection assays of broad specificity is likely to prove challenging. The analysis of the only published GVT-specific primer pair [24] suggests that it might miss some GVT isolates and that further development efforts are likely needed. The complete or near complete GVT genomic sequences reported here should prove an invaluable resource for such efforts. In this respect, a cursory look at the sequence alignments indicates that the use of degenerate primers could be the most interesting and fruitful strategy.

Due to its recent discovery, GVT is still a very poorly known virus. In particular, there are currently no indications about is potential impact in grapevine. Some of the plants analysed here, such as the French Sauvignon samples, the Slovak Welshriesling or the German Riesling did not show any symptoms or evidence of viral infection. This suggests that similar to GRSPaV, GVT might symptomlessly infect at least some grapevine varieties. Whether this notion can be further extended remains to be investigated.

## Supporting information

S1 Fig Phylogenetic tree reconstructed using the amino acid sequence of the replication-associated protein (REP) of GVT and of GRSPV isolates. The Neighbor joining tree was reconstructed using a strict amino acid distance in Mega7. Bootstrap analysis (1,000 replicates) was performed to evaluate the solidity of branches. Only bootstrap values >70% are shown. The scale bar represents 10% amino acid divergence. Groups of GRSPaV or GVT are indicated on the right of the Figure.
(TIF)

S2 Fig Phylogenetic tree reconstructed using the amino acid sequence of the coat protein (CP) proteins of GVT and of RSPV isolates. The Neighbor joining tree was reconstructed using a strict amino acid distance in Mega7. Bootstrap analysis (1,000 replicates) was performed to evaluate the solidity of branches. Only bootstrap values >70% are shown. The scale bar represents 10% amino acid divergence. Groups of GRSPaV or GVT are indicated on the right of the Figure.
(TIF)

S1 Text Sequences of the GVT isolates described in the present work. The sequences are additionally available in Genbank under the accession numbers provided in the text and tables.
(TXT)

## References

[1] Martelli GP. An overview on grapevine viruses, viroids, and the diseases they cause In: Meng B, Martelli GP, Golino DA, Fuchs M. editors. Grapevine Viruses: Molecular Biology, Diagnostics and Management. Springer International Publishing; 2017 pp 31–46.

[2] Massart S, Olmos A, Jijakli H, Candresse T. Current impact and future directions of high throughput sequencing in plant virus diagnostics. Virus Res. 2014; 188:90–96. 10.1016/j.virusres.2014.03.029 24717426

[3] Martelli GP, Jelkmann W. Foveavirus, a new plant virus genus. Arch Virol. 1998; 143:1245–1249. 9687881 10.1007/s007050050372

[4] Adams MJ, Candresse T, Hammond J, Kreuze JF, Martelli GP, Namba S, et al. Family Betaflexiviridae. In: King AMQ, Adams MJ, Carstens EB, Lefkowitz EJ editors. Virus Taxonomy—Ninth Report on the International Committee on Taxonomy of Viruses. Elsevier Academic Press, Cambridge, MA, USA, 2012, pp. 920–941.

[5] Mann K, Meng B. The triple gene block movement proteins of a grape virus in the genus Foveavirus confer limited cell-to-cell spread of a mutant Potato virus X. Virus Genes. 2013; 47:93–104. 10.1007/s11262-013-0908-0 23543158

[6] Alabi OJ, Martin RR, Naidu RA. (2010) Sequence diversity, population genetics and potential recombination events in grapevine rupestris stem pitting-associated virus in Pacific North-West vineyards. J Gen Virol. 2010; 91:265–276. 10.1099/vir.0.014423-0 19759241

[7] Nolasco G, Santos C, Petrovic N, Teixeira Santos M, Cortez I, Fonseca F, et al Rupestris stem pitting associated virus isolates are composed by mixtures of genomic variants which share a highly conserved coat protein. Arch Virol. 2006; 151:83–96. 10.1007/s00705-005-0611-0 16132183

[8] Terlizzi F, Ratti C, Filippini G, Pisi A, Credi R. Detection and molecular characterization of Italian Grapevine rupestris stem pitting-associated virus isolates. Plant Pathol. 2010; 59:48–58.

[9] Meng B, Rowhani A. Grapevine rupestris stem pitting-associated virus in: Meng B, Martelli GP, Golino DA, Fuchs M. editors. Grapevine Viruses: Molecular Biology, Diagnostics and Management. Springer International Publishing; 2017, pp 257–287.

[10] Meng B, Johnson R, Peressini S, Forsline PL, Gonsalves D. RSPaV-1 is consistently detected in rupestris stem pitting-infected grapevines. Eur J Plant Pathol. 1999; 105:191–199.

[11] Nakaune R, Inoue K, Nasu H, Kakogawa K, Nitta H, Imada J, et al Detection of viruses associated with rugose wood in Japanese grapevines and analysis of genomic variability of Rupestris stem pitting-associated virus. J Gen Plant Pathol. 2008; 74:156–163.

[12] Nolasco G, Mansinho A, Teixeira Santos M, Soares C, Sequeira Z, Sequeira C, et al (2000) Large scale evaluation of primers for diagnosis of rupestris stem pitting associated virus-1. Eur J Plant Pathol. 2000; 106:311–318.

[13] Zhang YP, Uyemoto JK, Golino DA, Rowhani A. Nucleotide Sequence and RT-PCR Detection of a virus associated with grapevine rupestris stem-pitting disease. Phytopathology. 1998; 88:1231–1237. 10.1094/PHYTO.1998.88.11.1231 18944859

[14] Meng B, Gonsalves D. 2003 Rupestris stem pitting-associated virus of grapevines: Genome structure, genetic diversity, detection, and phylogenetic relationship to other plant viruses. Curr Topics Virol. 2003; 3:125–135.

[15] Meng B, Zhu HY, Gonsalves D. Rupestris stem pitting associated virus-1 consists of a family of sequence variants. Arch Virol. 1999; 144:2071–2085. 10603163 10.1007/s007050050623

[16] Mostert I, Burger JT, Maree HJ. Genetic diversity and identification of putative recombination events in grapevine rupestris stem pitting-associated virus. Arch Virol. 2018; 163:2491–2496. 10.1007/s00705-018-3883-x 29796924

[17] Glasa M, Predajna L, Soltys K, Sihelska N, Nagyova A, Wetzel T, et al Analysis of Grapevine rupestris stem pitting-associated virus in Slovakia Reveals Differences in Intra-Host Population Diversity and Naturally Occurring Recombination Events. Plant Pathol J. 2017; 33:34–42. 10.5423/PPJ.OA.07.2016.0158 28167886 PMC5291396

[18] Hily JM, Beuve M, Vigne E, Demangeat G, Candresse T, Lemaire O. A genome-wide diversity study of *Grapevine rupestris stem pitting-associated* virus. Arch Virol. 2018; Epub ahead of print, 10.1007/s00705-018-3945-0 30043203

[19] Terlizzi F, Li C, Ratti C, Qu W, Credi R, Meng B. Detection of multiple sequence variants of Grapevine rupestris stem pitting-associated virus using primers targeting the polymerase domain and partial genome sequencing of a novel variant. Ann Appl Biol. 2011; 159:478–490.

[20] Massart S, Candresse T, Gil J, Lacomme C, Predajna L, Ravnikar M, et al (2017) A Framework for the Evaluation of Biosecurity, Commercial, Regulatory, and Scientific Impacts of Plant Viruses and Viroids Identified by NGS Technologies. Front Microbiol. 2017; 8:45, 10.3389/fmicb.2017.00045 28174561 PMC5258733

[21] Jo Y, Song MK, Choi H, Park JS, Lee JW, Lian S, et al Genome Sequence of Grapevine Virus T, a Novel Foveavirus Infecting Grapevine. Genome Announc. 2017; 5, e00995–17. 10.1128/genomeA.00995-17 28912330 PMC5597771

[22] Ruiz-Garcia AB, Okic A, Nourinejhad Zarghani S, Olmos A, Wetzel T. First report of grapevine virus T in grapevine in Germany. Plant Dis. 2018; 102:1675.

[23] Voncina D, Almeida RPP. Screening of some Croatian autochthonous grapevine varieties reveals a multitude of viruses, including novel ones. Arch Virol. 2018; 163:2239–2243. 10.1007/s00705-018-3850-6 29680925

[24] Glasa M, Predajna L, Sihelska N, Soltys K, Ruiz-Garcia AB, Olmos A, et al Grapevine virus T is relatively widespread in Slovakia and Czech Republic and genetically diverse. Virus Genes. 2018; Epub ahead of print, 10.1007/s11262-018-1587-7 29995199

[25] Marais A, Faure C, Bergey B, Candresse T. Viral double-stranded RNAs (dsRNAs) from plants: alternative nucleic acid substrates for high-throughput sequencing. In: Pantaleo V, Chiumenti M. editors Viral metagenomics: methods and protocols, Methods Molec Biol. 2018; 1746:45–53. 10.1007/978-1-4939-7683-6_4 29492885

[26] Candresse T, Marais A, Faure C, Gentit P. Association of Little cherry virus 1(LChV1) with the Shirofugen stunt disease and characterization of the genome of a divergent LChV1 isolate. Phytopathology. 2013; 103:293–298. 10.1094/PHYTO-10-12-0275-R 23402630

[27] Altschul SF, Gish W, Miller W, Myers EW, Lipman DJ. Basic Local Alignment Search Tool. J Mol Biol. 1990; 215:403–410. 10.1016/S0022-2836(05)80360-2 2231712

[28] Thompson J, Higgins DG, Gibson TJ. CLUSTALW: improving the sensitivity of progressive multiple sequence alignment through sequence weighting, position-specific gap, penalties and weight matrix choice. Nucleic Acids Res. 1994; 22:4673–4680. 7984417 10.1093/nar/22.22.4673 PMC308517

[29] Kumar S, Stecher G, Tamura K. MEGA7: Molecular Evolutionary Genetics Analysis Version 7.0 for Bigger Datasets. Mol Biol Evol. 2016; 33:1870–1874. 10.1093/molbev/msw054 27004904 PMC8210823

[30] Librado P, Rozas J. DnaSP v5: a software for comprehensive analysis of DNA polymorphism data. Bioinformatics 2009; 25:1451–1452. 10.1093/bioinformatics/btp187 19346325

[31] Martin DP, Murrell B, Golden M, Khoosal A, Muhire B. RDP4: Detection and analysis of recombination patterns in virus genomes. Virus Evol. 2015; 1:1–5. 27774277 10.1093/ve/vev003 PMC5014473

[32] Weaver S, Shank SD, Spielman SJ, Li M, Muse SV, Kosakovsky Pond S.L. Datamonkey 2.0: A Modern Web Application for Characterizing Selective and Other Evolutionary Processes. Mol Biol Evol. 2018; 35:773–777. 10.1093/molbev/msx335 PMC5850112 29301006

[33] Teycheney PY, Laboureau N, Iskra-Caruana ML, Candresse T. High genetic variability and evidence for plant-to-plant transfer of Banana mild mosaic virus. J Gen Virol. 2005; 86:3179–3187. 10.1099/vir.0.81197-0 16227242

[34] Hu GJ, Dong YF, Zhu HJ, Zhang ZP, Fan XD, Ren F, et al Molecular characterizations of two grapevine rupestris stem pitting-associated virus isolates from China. Arch Virol. 2015; 160:2641–2645. 10.1007/s00705-015-2544-6 26215445

