# Supplementary figures and images for: Grapevine virus T diversity as revealed by full-length genome sequences assembled from high-throughput sequence data
Source: PLoS One. 2018 Oct 30;13(10):e0206010. doi: 10.1371/journal.pone.0206010 (PMC6207325; doi:10.1371/journal.pone.0206010)

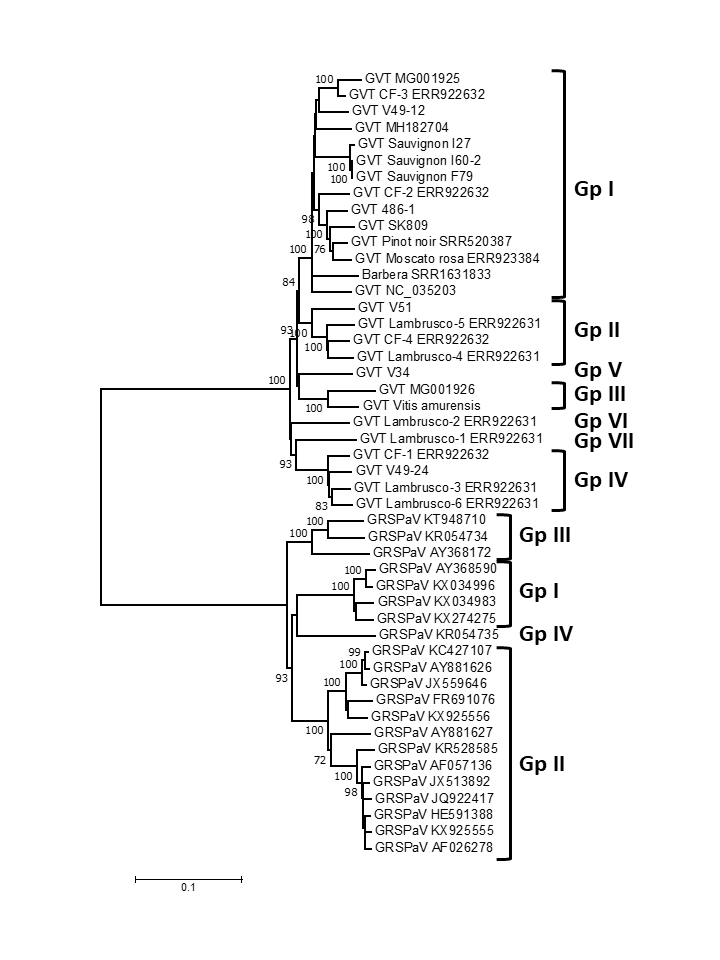

Supplement: S1 Fig — The Neighbor joining tree was reconstructed using a strict amino acid distance in Mega7. Bootstrap analysis (1,000 replicates) was performed to evaluate the solidity of branches. Only bootstrap values >70% are shown. The scale bar represents 10% amino acid divergence. Groups of GRSPaV or GVT are indicated on the right of the Figure. (TIF) [file pone.0206010.s001.tif]

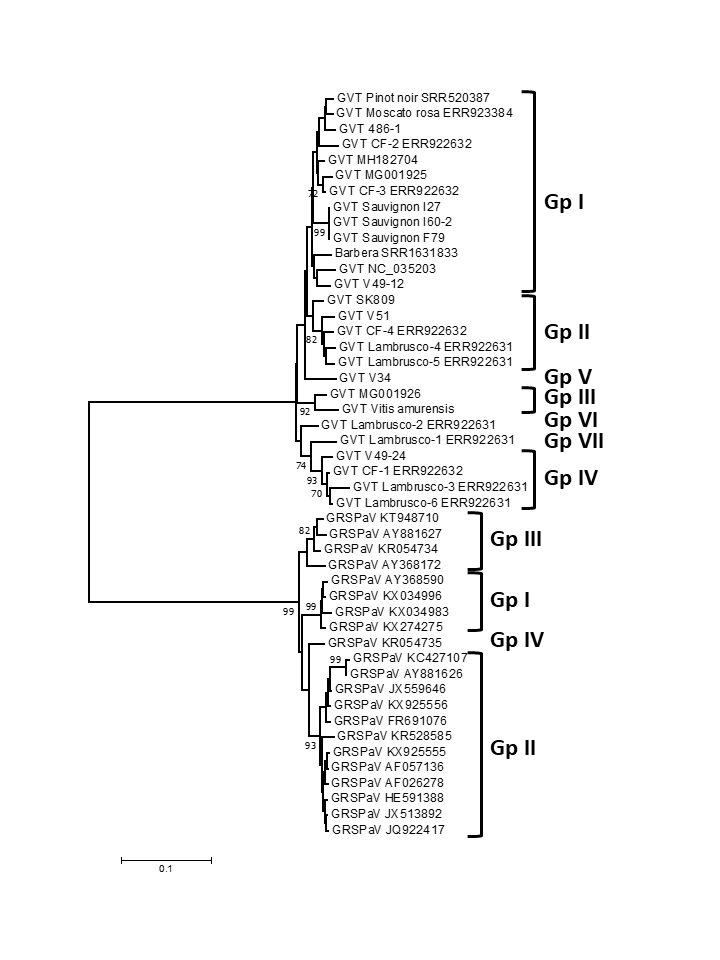

Supplement: S2 Fig — The Neighbor joining tree was reconstructed using a strict amino acid distance in Mega7. Bootstrap analysis (1,000 replicates) was performed to evaluate the solidity of branches. Only bootstrap values >70% are shown. The scale bar represents 10% amino acid divergence. Groups of GRSPaV or GVT are indicated on the right of the Figure. (TIF) [file pone.0206010.s002.tif]
